# Evidence for a common mechanism of spatial attention and visual awareness: Towards construct validity of pseudoneglect

**DOI:** 10.1371/journal.pone.0212998

**Published:** 2019-03-07

**Authors:** Jiaqing Chen, Jagjot Kaur, Hana Abbas, Ming Wu, Wenyi Luo, Sinan Osman, Matthias Niemeier

**Affiliations:** 1 Department of Psychology, University of Toronto, Toronto, Ontario, Canada; 2 Centre for Vision Research, York University, Toronto, Ontario, Canada; University of Plymouth, UNITED KINGDOM

## Abstract

Present knowledge of attention and awareness centres on deficits in patients with right brain damage who show severe forms of inattention to the left, called spatial neglect. Yet the functions that are lost in neglect are poorly understood. In healthy people, they might produce “pseudoneglect”—subtle biases to the left found in various tests that could complement the leftward deficits in neglect. But pseudoneglect measures are poorly correlated. Thus, it is unclear whether they reflect anything but distinct surface features of the tests. To probe for a common mechanism, here we asked whether visual noise, known to increase leftward biases in the grating-scales task, has comparable effects on other measures of pseudoneglect. We measured biases using three perceptual tasks that require judgments about size (landmark task), luminance (greyscales task) and spatial frequency (grating-scales task), as well as two visual search tasks that permitted serial and parallel search or parallel search alone. In each task, we randomly selected pixels of the stimuli and set them to random luminance values, much like a poor TV signal. We found that participants biased their perceptual judgments more to the left with increasing levels of noise, regardless of task. Also, noise amplified the difference between long and short lines in the landmark task. In contrast, biases during visual searches were not influenced by noise. Our data provide crucial evidence that different measures of perceptual pseudoneglect, but not exploratory pseudoneglect, share a common mechanism. It can be speculated that this common mechanism feeds into specific, right-dominant processes of global awareness involved in the integration of visual information across the two hemispheres.

## Introduction

The term pseudoneglect refers to a set of intact functions of spatial attention and perceptual awareness in healthy people that feature small but robust leftward biases [1–3] and that are thought to complement some of the left-sided deficits that patients exhibit after right-brain damage, called spatial neglect [4, 5]. However, using a neuropsychological syndrome is a rather unsatisfactory way to delineate intact functions. It shows how little is known about functions underlying pseudoneglect. As we will argue in the following this is a problem of unclear validity of pseudoneglect research data.

First of all, because pseudoneglect research is importantly based on neuropsychological data it relies heavily on face validity. For example, the line bisection task, a clinical paper-pencil test that measures rightward biases in patients, is believed to capture left-biased pseudoneglect in neurologically healthy people as well (e.g., [1–3]]. Furthermore, the line bisection task has inspired a variety of perceptual judgment tasks where the left and right side of visual stimuli are compared with respect to different features such as horizontal width, luminance, numerosity, spatial frequency, or halves of chimeric faces [4–15]. Another example are tasks that are believed to find pseudoneglect when participants are required to detect objects in random search arrays (for a comprehensive review, e.g., [16]) or at one or two lateral positions [17–19], and with or without attentional cues ([20]; but see [21] for no lateralization effect of cueing). In addition, non-visual tasks such as perceptual judgments of tactile and auditory stimuli [3,22] as well as tests that probe mental representations and imagery ([23–31]; for a review: [32]) also produce left-biased performance, opposite to what is observed in neglect in similar experiments.

However, such paradigmatic similarity to neuropsychological studies in patients together with complementary leftward biases are validation criteria that come with important limitations. Paradigmatic similarity is an insufficient criterion because neglect and pseudoneglect tests typically allow for a large range of task strategies so that it is quite possible that patients and healthy individuals employ entirely different strategies that trigger different neural mechanisms. Hence, it is risky to assume that the same test measures the same construct in different populations of participants with fundamentally different cognitive profiles. As for complementary task results, left-biased performance might neither be a necessary nor a sufficient criterion. Leftward biases might be (at least in part) not necessary, because deficits observed in neglect include non-lateralized dysfunctions [33]. On the other hand, a leftward bias is not a sufficient criterion because healthy people show various biases and lateralized mechanisms that are not related to attention and awareness. For example, healthy people are better at grasping objects with two hands in their left than right visual field [34, 35] due to a right-brain dominance [36, 37] that is not related to spatio-attentional functions [34]. Thus, validating pseudoneglect as a construct merely based on its similarity to neuropsychological research would be insufficient.

Better validation, i.e., criterion validity, can be achieved when pseudoneglect measures turn out to be sensitive to certain experimental manipulations. For example, attentional cues [38–40], distractors [41], reduced alertness [6, 18, 42], or increased cognitive load [43] have been reported to influence pseudoneglect measures. Looking at these reports together, it is tempting to conclude that, collectively, they appear to be consistent with the idea that pseudoneglect reflects a system of spatial functions of attention and awareness. However, combining and generalizing research results in such a way (and thereby assuming construct validity) might be imprudent. It hinges on the faulty assumption that the different effects relate to the same measure of pseudoneglect although the effects were obtained with different pseudoneglect paradigms.

Unfortunately however, the different pseudoneglect paradigms are not statistically related with one another: Only a few studies report correlations between different visual ([10]; for some conditions: [14]) and non-visual measures of pseudoneglect [22, 44]. Most studies find non-significant correlations ([4, 9, 11–13, 45, 46]; one study even reported line bisection and the grating-scales task to be negatively correlated [45], note though that a suboptimal version of the latter task was used, also see Methods).

Crucially, Learmonth and colleagues [45] conducted a sizeable study in which they administered five pseudoneglect tasks within the same group of participants twice across two days. Despite good test-retest reliability, inter-test correlations were weak, and a principal component analysis gave rise to a component structure where all but one extracted component explained variance in just one pseudoneglect task. This result allows for two possible interpretations: the tests might measure similar mechanisms of attention and awareness but their effect sizes are too small relative to less interesting task-specific processes and strategies so that covariances disappear. Alternatively, the different pseudoneglect tasks might reflect different kinds of true pseudoneglect biases to do with different mechanisms underlying attention and awareness.

Such different forms of pseudoneglect could mirror evidence for different subtypes of spatial neglect [33, 47, 48] and related deficits such as extinction [49–51]. Indeed, studies comparing patient performance across multiple clinical tests have reported poor correlations ([7, 51]; but see [52]) and multiple factors [53–57]. Together these studies seem to point at independently impaired processes such as allocentric or object-based deficits [57], representational neglect [54], perceptual forms of neglect [55–57], and exploratory/ visuomotor impairments [56, 57].

However, projecting these clinical findings back onto data in the intact brain comes with several caveats. First, clinical tests are often administered as paper-and-pencil tests in, at times, severely affected individuals, thereby creating risks of multiple motor, perceptual, and cognitive confounds. Second, correlations in patient performance are influenced by the statistical patterns of the patients’ brain damages. That is, because lesions due to stroke, the most common etiology in neglect, follow the regularities of the cerebro-vascular system, the non-random patterns of brain damage add covariance that distorts correlative structures among cognitive dysfunctions. A third caveat is that the correlation studies in neglect have used large varieties of paradigms. In contrast, studies in pseudoneglect heavily focus on one particular group of tests, i.e., perceptual judgment tasks [4, 9–14, 45, 46], which makes the absence of correlations among them all the more mysterious.

**Fig 1:**
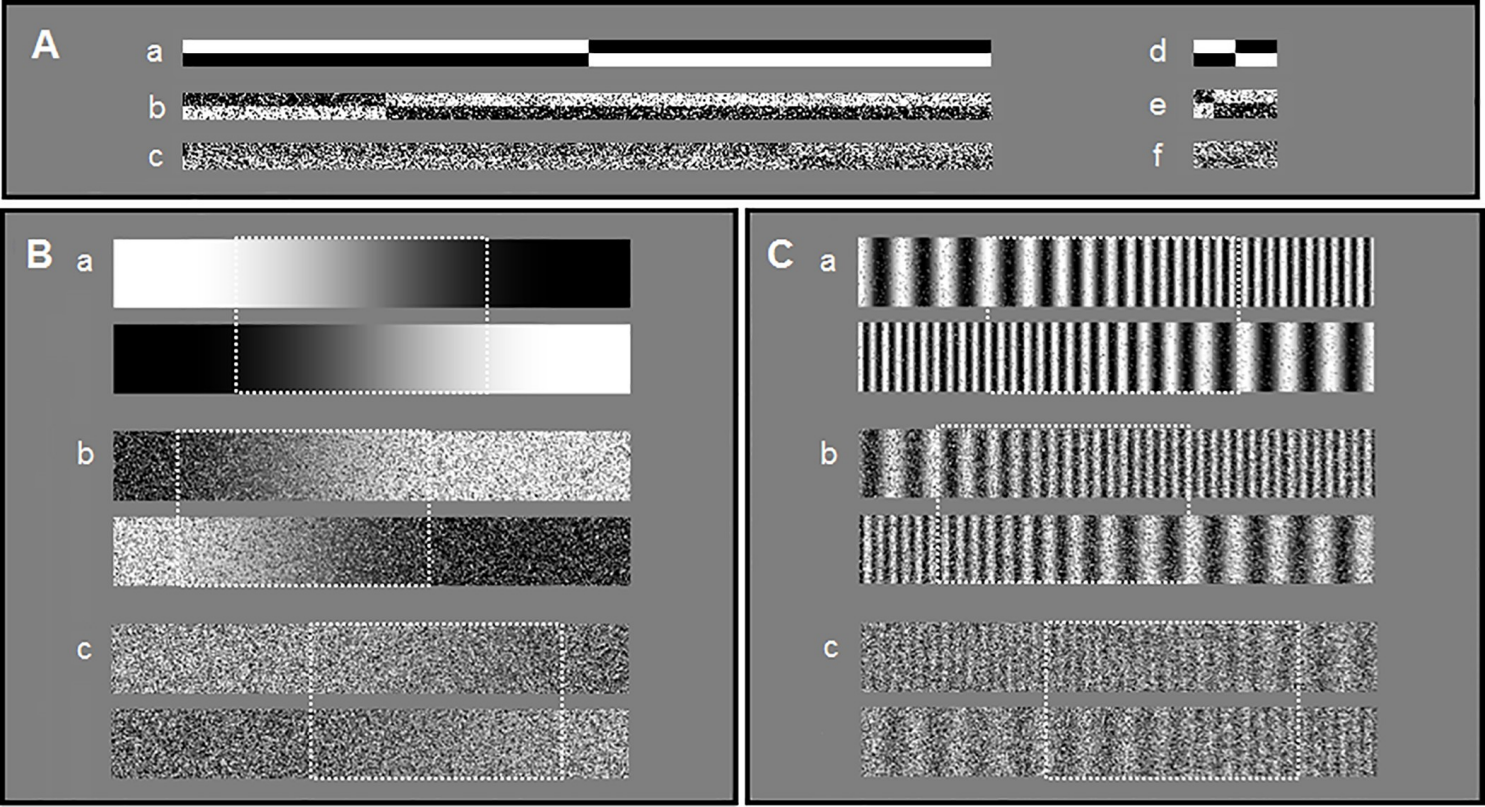
Samples of the landmark, greyscales, and grating-scales stimuli. (A) Stimuli a-c are examples of long lines (20° × 0.68°) and stimuli d-f are examples of short lines (2° × 0.68°). Stimuli a and d: 0% asymmetry, 0% noise; Stimuli b and e: -25% asymmetry, 42% noise; Stimuli c and f: 25% asymmetry, 84% noise. (B) White dashed boxes (not presented during task) indicate the area in which luminance changed from white to black and vice versa. Stimulus a: 0% asymmetry, 0% noise; Stimulus b: -20% asymmetry, 42% noise; Stimulus c: 20% asymmetry, 84% noise. (C) White dashed boxes (not presented during task) indicate the area in which gratings transformed from low spatial frequency to high spatial frequency and vice versa. Stimulus a: 0% asymmetry, 7% noise; Stimulus b: -20% asymmetry, 42% noise; Stimulus c: 20% asymmetry, 84% noise. All stimuli in (B) and (C) are 20° wide, 2.76° tall.

To complicate matters, even the few correlations that exist among pseudoneglect task biases are not necessarily easy to interpret. For example, the biases observed in the grating-scales task and its control condition ([14, 58]; see Fig 1 and Methods for details) correlate positively [40, 59, 60], but only the former bias is a leftward bias whereas the latter is a rightward bias. This suggests that the grating-scales task must involve more than one biasing mechanism [59] with only the experimental, but not the control condition of the task interacting with attentional cues [40]. Despite these qualitative differences, the two task conditions would arguably load on the same factor. Thus factors can quite possibly reflect functions that are not related to attention and awareness.

In sum, understanding pseudoneglect is hampered by a lack of validation of its measures. This includes mere investigation of face validity, overgeneralization of criterion validity, and, to date, incomplete success of attempts to establish construct validity through correlative approaches.

Here we chose an alternative approach. Rather than comparing pseudoneglect tasks by administering them to the same group of participants, we applied the same experimental manipulation across different tasks. Specifically, we used pixel noise to impoverish test stimuli, much like a TV image with poor reception. Previous studies have shown that pixel noise amplifies leftward biases in the experimental condition of the grating-scales task as well as increases the difference between its experimental and its control condition [59–61] for reasons that have yet to be investigated in more detail. So far it is clear that the pixel noise effect occurs with high-contrast, luminance-defined stimuli as well as with stimuli that are isoluminant which indicates that the noise effect could arise within the parvocellular system alone ([61], although a separate contribution of the magnocellular system is possible: [62, 63]). Further, no pixel noise effect is observed in individuals with ADHD where cognitive distraction would cause mild symptoms of neglect [60]. Therefore, rather than causing cognitive distraction, the pixel noise effect appears to be a form of perceptual activation of earlier visual areas where neurons with small receptive fields respond more strongly to images corrupted by visual noise at the expense of higher-order visual information [64, 65]. As a result, the increased response of the early visual system itself might then have an asymmetrical effect down-stream [60]. That is, a downstream right-brain dominant system could be exponentially activated through iterative feed-forward and feedback loops and/or through stronger interhemispheric competition with non-dominant areas in the left brain [17, 71, 19].

So far, the pixel noise effect has been only tested in the grating-scales task paradigm (although see [66], who used pixel noise on one side of line bisection items). Thus, it remains unclear whether pixel noise targets processes that are specific to the grating-scales task or that are involved in other tasks as well. Therefore, investigating the effect of pixel noise across different tasks can help identify potential groups of pseudoneglect tasks that test similar processes and/or distinguish between different forms of pseudoneglect, so as to strive for construct validity in the form of convergent and discriminant validity.

To attain convergent and discriminant validity for pseudoneglect tests, in the present study we asked whether comparable noise effects occur in tasks other than the grating-scales task. Specifically, we tested the effects of different levels of pixel noise in three perceptual judgment tasks: landmark task [67], greyscales task [12], and grating-scales task [14] as well as two visual search tasks [16]. Our main aim was to test whether pseudoneglect biases as observed in these tasks would become more pronounced with pixel noise while, secondly, ensuring that biases did not increase for trivial reasons such as noise making tasks more difficult. For that reason we included control conditions wherever possible.

## Methods

### Participants

One hundred and fifteen undergraduate students from the University of Toronto at Scarborough gave their informed and written consent prior to the experiment and obtained a course credit. As pre-planned based on a pilot landmark experiment we recruited participant groups sized in the low 20’s for the perceptual judgment tasks (also see a power analysis conducted in [60]): Twenty-two students participated in the landmark task (11 males; mean age = 19.1 years, SD = 2.3 years), 21 participated in the greyscales task (6 males; mean age = 20.1 years, SD = 5.0 years), and 22 participated in the grating-scales task (7 males; mean age = 21.3, SD = 4.6). Our two visual search experiments tested 25 participants each (standard visual search task: 9 males; mean age = 18.8 years, SD = 1.8 years; masked visual search task: 5 males; mean age = 20.1 years, SD = 3.1 years) to exceed the number of participants of the experiment after which our standard search task was modelled (first experiment in, reference [16]). All experimental procedures were approved by the Human Participants Review Sub-Committee of the University of Toronto and have therefore been performed in accordance with the ethical standards laid down in the 1964 Declaration of Helsinki. All participants were free of neurological diseases and psychiatric disorders with normal or corrected-to-normal vision, and all were right-handed as confirmed by the Edinburgh handedness inventory [68].

### Stimuli and procedure

Participants sat at a table with their chin placed on a chin rest to keep head movements to a minimum. Sixty centimetres in front of and aligned with the head was a 19” CRT monitor (Viewsonic; 100 Hz) on which we presented five different tasks programmed in Matlab with the Psychophysics Toolbox extension [69, 70]. Trials of all tasks began with a fixation phase (500–1000 ms) where a green square (0.26° across) appeared in the centre of a middle-grey background (about 26.8 cd/m^2^). Participants were asked to fixate the square with their eyes and maintain fixation throughout the trials. Eye movements were not recorded but ocular scanning of stimuli was essentially impossible because stimuli were presented too briefly. Presentation time for stimuli of perceptual judgment tasks was 80 ms (greyscales and grating-scales) or 150 ms (landmark stimuli) to approximately equate levels of difficulty. Visual search arrays appeared for 150 ms as well. All tasks we used pixel noise as an independent variable, that is, we would randomly select certain proportions of stimulus pixels (e.g., for the Landmark task we would select 0% or 42%, or 84% of all the pixels of the Landmark stimulus etc.) and set them to random levels of luminance (i.e., to any of the 256 possible grey levels). Trials ended with a grey response screen, and participants responded with a key press.

#### Landmark task [67, 71]

We used a version of the task adapted from McCourt [72] that presents horizontal lines (0.68° thick; 20° or 2° wide) on a middle grey background with all lines consisting of black and white rectangles so to create two segments with a contrast-defined vertical transection (Fig 1A). Transections could appear anywhere along the lines between +/-25% from right- or leftward relative to horizontal stimulus width (e.g., Fig 1A: b-c and 1A: e-f). We asked participants to press the arrow-left or arrow-right key on the number pad to indicate whether they felt that the left or the right line segment was shorter [8]. For their responses we made sure that participants could not use the fixation point as a reference because all stimuli were slightly shifted away from screen centre (6 visual degrees to the left or right, and up or down from centre). Responses were then used to estimate the dependent variables, bias and slope, with an adaptive procedure (see below, Psychophysical Methods section). The first independent variable was Noise (i.e., 0%, 42%, or 84% of random pixels of each stimulus were set to a random luminance value between white and black from trial to trial). Noise level varied from trial to trial. The second independent variable was Line length (20° long vs. 2° short lines). Long lines are known to produce left-biased pseudoneglect, but short lines are not, sometimes yielding cross-over [5, 73]. Biases obtained with short lines might reflect, in part, different mechanisms than biases obtained with long lines ([73]; although see [74]). Therefore, here we included the short lines as a control condition so as to gauge the specificity of a possible noise effect on the landmark task. There were two long-line and two short-line blocks (264 trials each) that were presented in an ABBA or BAAB order and that were preceded by 10 practice trials.

#### Greyscales task [12, 13]

For the current experiment, we used a modified version of the task suitable to measure psychometric functions [14]. Task stimuli consisted of pairs of horizontal bars (20° × 2.76°) on a middle grey background (Fig 1B). Luminance within each bar changed smoothly from black to white and white to black as a function of a half-cycle of a cosine within an approximately central area (dashed rectangles in Fig 1B), which could be located anywhere between +/-20% from right to left relative to the width of the bars (e.g., Fig 1B: b-c). The bars’ left and right ends remained constant in luminance so that both bars ranged from black to white and vice versa in all trials. After each trial, participants pressed the arrow-up or arrow-down key on the number pad depending on which of the two bars, to them, appeared darker on average. We then converted these responses into choices of bars with black sides on the left and right side (note that this approach disentangles perceptual and response biases; [12]). The responses were used to estimate the dependent variables, bias and slope, with an adaptive procedure equivalent to the one used for the landmark task (see below, Psychophysical Methods section). Again there was the same independent variable of Noise (0%, 42%, or 84% pixel noise). To our knowledge, the greyscales task has no control condition comparable to the short-line condition of the landmark task. Participants completed 10 practice trials and then four experimental blocks of 264 trials each.

#### Grating-scales task (GST, [14])

The task was inspired by the greyscales task [4, 12, 13] and its stimuli are designed similarly to the greyscales bars as described above except that the task feature is spatial frequency instead of luminance. That is, the GST presents pairs of horizontal bars (20° × 2.76°) filled with luminance-defined sine wave gratings that increase in spatial frequency from low to high levels (0.6 to 2 cycles per degree, called the “G2” stimulus) leftward and rightward in the upper and lower bar, respectively, or vice versa (Fig 1C). Frequency increases smoothly as a function of a half-cycle of a cosine within an approximately central area, which could be located anywhere between +/-20% from right to left relative to the width of the bars (e.g., Fig 1C: b-c) with the bars’ left and right ends remaining constant in spatial frequency so that all stimuli span the same range of frequencies (note that this is the “continuous version” of the grating-scales task, introduced in Experiment 1C in [14]; other experiments in the same study, also see [45], used stimuli with step-wise increases in spatial frequency with the risk that participants noticed the abrupt frequency increases and employed alternative task strategies).

Participants performed two versions of the GST. In the experimental condition or “high spatial frequency condition” they pressed the arrow-up or arrow-down key on the number pad depending on “which of the two bars had more of the thinner stripes”. In the control condition or “low spatial frequency condition” they responded depending on “which of the two bars had more of the thicker stripes.” We have previously shown that high and low spatial frequency instructions yield qualitatively different perceptual biases [40, 59] and electrophysiological correlates [75], arguably due to differences in perceptual salience [40, 76]. To determine people’s individual biases we converted their choices of upper and lower bars into choices of bars where the high spatial frequency component appeared on the left and right side, and we submitted the choices to an adaptive procedure just like for the other two perceptual judgment tasks, thus, yielding psychometric functions whose biases, as well as slopes, served as our dependent variables (see below, Psychophysical Methods section). Independent variables were Noise (levels: 0%, 42%, and 84%) and Instructions (high vs. low spatial frequency conditions).

#### Psychophysical methods

For the three perceptual judgments tasks, we mapped people’s probability to make “left” or “right” decisions (reporting the left or right part of landmark stimuli to be shorter, or choosing the greyscales or grating-scales bar with the darker or higher spatial frequency component on the left or right) as a function of stimulus asymmetry. These responses are well described by sigmoid functions. Here we used cumulative Gaussians with two free parameters: (1) point of subjective equality (PSE, i.e., the degree of stimulus asymmetry where a participant makes “left” and “right” responses equally often) that measures a person’s perceptual bias or pseudoneglect, and (2) slope (i.e., the rate at which the sigmoid function transitions from “left” to “right” responses or vice versa) that measures sensitivity or task difficulty (also see [14]). To estimate the two parameters for a given participant and condition, we used Matlab’s nlinfit function to iteratively search for the two parameter values that would produce the best fitting cumulative Gaussian to describe the participant’s data. This procedure works best if the person is tested with sufficient numbers of test trials that present stimuli whose asymmetry is near the PSE [77]. Therefore, here we use a simple adaptive testing procedure. The procedure begins with 10 test trials where stimulus asymmetries are chosen at random (+/-20% for the greyscales task and the GST; +/-25% given the somewhat more difficult short-line condition of the landmark task). After that, the procedure uses, trial by trial, all data collected so far to fit a preliminary cumulative Gaussian and, thus, obtain an estimate of the PSE. This estimate then determines how asymmetrical the stimulus should be for the next trial. However, only half of the time this asymmetry is actually used. The other half of trials uses a stimulus with the opposite asymmetry. That is, across a block of trials stimuli are not systematically skewed to the left or right so as to prevent perceptual or motor adaptation to certain asymmetries, or cognitive strategies [78]. Nevertheless, in pilot tests we found that this adaptive procedure requires at most half of the number of trials compared to the method of constant stimuli to attain comparable levels of accuracy.

#### Standard visual search task [16]

**Fig 2:**
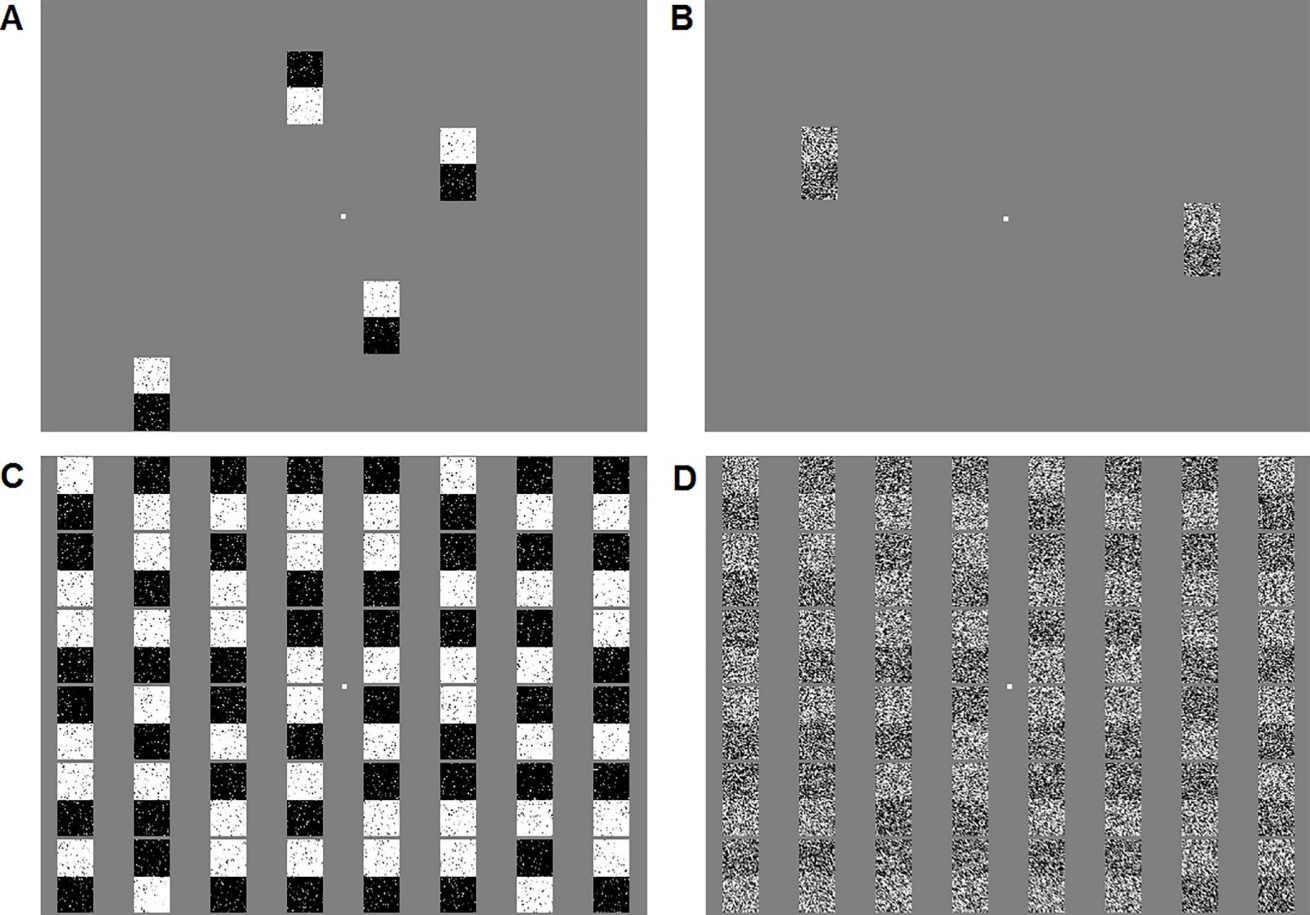
Samples of the visual search and masking stimuli. (A) An example of the visual search array when the target is present, noise is 7%, and set size is 4. (B) An example of the visual search array when the target is absent, noise is 84%, and set size is 2. (C) An example of the masking screen following 7% noise visual search array in the masked visual search task. (D) An example of the masking screen following 84% noise visual search array in the masked visual search task.

We chose a time-limited detection paradigm that, to our knowledge, is one of the most thoroughly studied paradigms with respect to reliably producing pseudoneglect, arguably, as a result of interhemispheric competition [16], regardless of reading habits [79]. (A recent study also used a target detection paradigm including a condition with similar time limitation, but found no evidence for pseudoneglect in that condition. However, as the authors pointed out their large set size might have made the condition too difficult; see [80], 200-ms condition in their Experiment 1.) Because pseudoneglect should occur for a wide range of search items [16], here we designed search items to resemble those of the perceptual judgment tasks. Specifically, the stimuli were designed to look like pieces of greyscales, i.e., they were rectangles (1.01° × 2.02°) made up of one black and one white square with either the white or the black square at the top (Fig 2A and 2B) and with one of these stimuli consistently serving as target and the other as distractors (counterbalanced across participants). Stimuli were superimposed with different amounts of pixel noise as described for the perceptual judgment experiments. In contrast to the latter experiments, however, we dropped the intermediate level of noise so as to optimize our chances of obtaining a noise effect on pseudoneglect. Secondly, we selected a different set of levels: 7% and 84% as opposed to 0% and 84% noise. Although both sets of levels are quite similar, the former ensured that noise solely varied in a quantitative manner whereas the latter would have varied noise quantitatively as well as categorically in terms of noise present vs. absent (note that this was unproblematic for the perceptual judgment experiments that tested three noise levels but might have contributed to the fact that pseudoneglect biases increased relatively little from 0% to intermediate noise levels, also see ref. [59–61]).

Search arrays appeared for 150 ms at locations randomly chosen from 48 possible locations (8 columns × 6 rows) with the same number of stimuli on the left and right side, amounting to set sizes of 2, 4, or 8 items as we were unsure whether certain sizes were more suitable to reveal a possible pixel noise effect on pseudoneglect (in pilot tests we found that searches with more than 8 items became very difficult and might have produced floor effects). Participants were asked to decide as quickly but also as accurately as possible whether a search array contained a target (“j” key) or not (“n” key; 50% probability). Dependent variables capturing search performance were percent correct trials and reaction times, calculated as medians of individual correct responses after trimming (responses faster than 100 ms or slower than four standard deviations above the individual mean reaction time, rejection rate = 1.49%, SD = 0.41; we also inspected means of individual reaction times and medians of individual reaction times without trimming but found these data to yield very similar results). Independent variables were Set size (2, 4, or 8 items), Pixel noise (7% vs. 84% pixels noise), and Visual field (left vs. right). A main effect of Set size was expected given the effortful nature of the searches. A main effect of Noise should reflect perceptual difficulty due to the poorer visibility of noisy search items and as such served to control whether pixel noise had any experimental effect on the searches equivalent to the effect of noise on the slopes of psychometric functions in the perceptual judgment experiments. Further, equivalent to the left-biased PSEs observed for the perceptual judgment tasks we expected a left visual field advantage in reflection of pseudoneglect, either across all set sizes (i.e., as main effect), or for specific set sizes (i.e., as a Visual field-by-Set size interaction). Crucially, the experiment was devised to test for a Noise-by-Visual field (or a Noise-by-Visual field-by-Set size) interaction; that is, the left visual field advantage should increase with pixel noise at least for certain set sizes if pixel noise amplified the attentional bias that underlies pseudoneglect in visual search tasks.

A sample of the respective target stimulus was shown to each participant at the start of each experiment, followed by 10 practice trials. Each participant completed four equal-sized blocks of 960 trials in total (3 set sizes * 2 noise levels * 2 visual fields * 40 target trials plus 3*2*80 no-target trials), thus, we administered the same number of trials as in [16] to avoid any time-on-task effects [42].

#### Masked visual search task

Because the standard visual search task (in contrast to the perceptual judgment experiments) found pseudoneglect to be unchanged by pixel noise (see Results), in a follow-up experiment we tested whether noise does influence pseudoneglect during visual search if the searches are predominantly parallel. To elaborate, visual searches often involve a mix of parallel and serial processes. We reasoned that (a) serial processes might have dominated in our standard search task despite the time limitations because participants could have covertly scanned their iconic memory, and (b) such mental scanning could have played a smaller role during our perceptual judgment experiments, given that these tasks show just one stimulus at any time. At least for the landmark task, it has been demonstrated that pseudoneglect biases remain unchanged when visual masks delete iconic memory and thus prevent mental scanning [5]. Therefore, we reasoned that perhaps noise influenced pseudoneglect in the current perceptual judgments tasks because it was predominantly based on parallel processes. If so, visual searches that are mainly parallel [81] might yield pseudoneglect that changes with noise as well.

To force participants to use mainly parallel forms of search, we used a masked visual search task where the masks served to disrupt iconic memory, thereby minimizing serial scanning. We repeated the visual search task with the same parameters, with two exceptions: Each search array was immediately followed by a mask (until response) that was composed of 48 target and distractor stimuli appearing at all 48 possible locations and with the same amount of noise as the search array (Fig 2C and 2D). The purpose of the mask was to minimize serial scanning of the array [81]. At the same time, the mask made the task more difficult. Therefore we dropped set size 8. After 10 practice trials, participants completed four blocks of 160 trials each, i.e., 640 trials in total (2 set sizes * 2 noise levels * 2 visual fields * 40 target trials plus 3*2*80 no-target trials).

## Results

**Fig 3:**
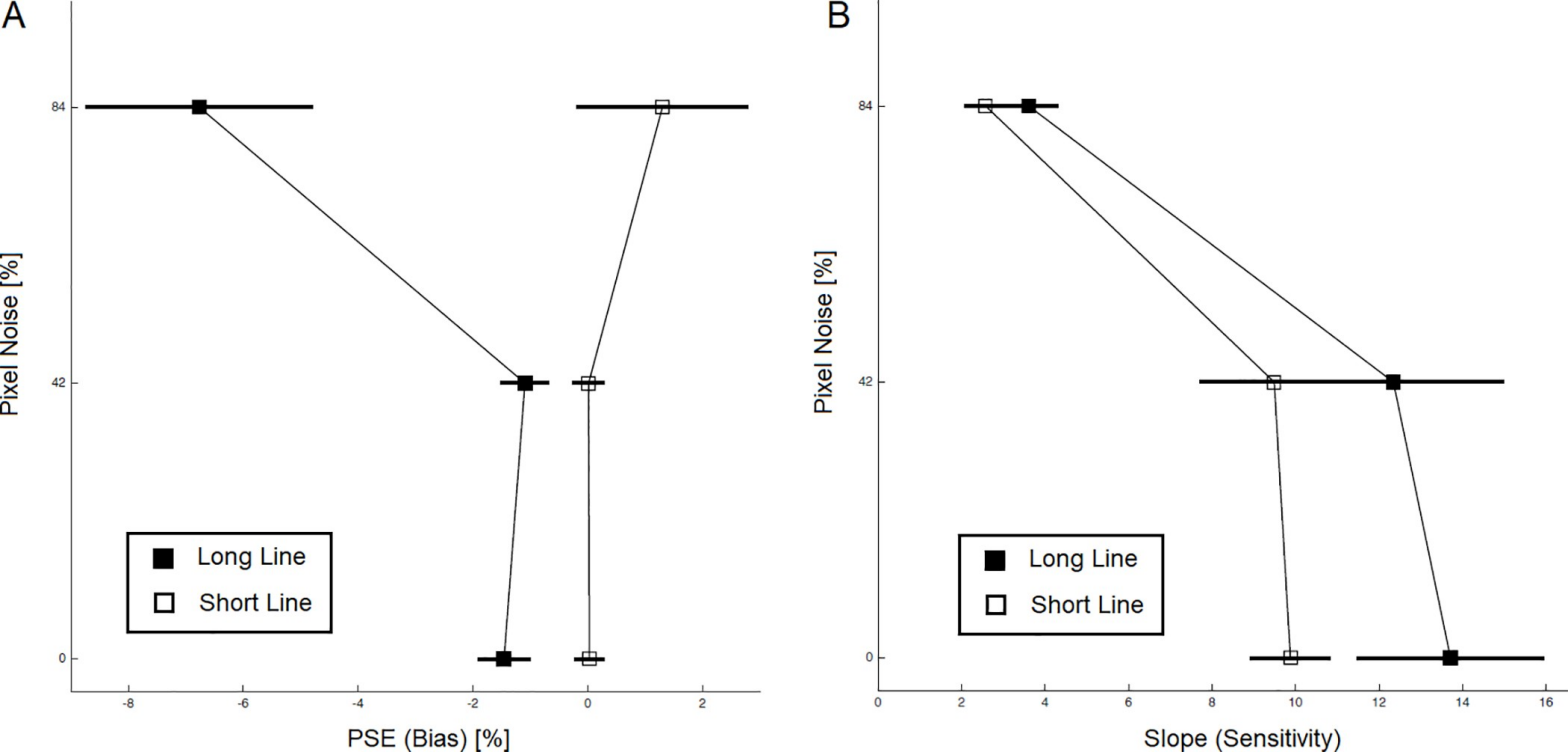
Results for the landmark experiment. (A) Group average of perceptual bias quantified as point of subjective equality (PSE) of the psychometric functions. (B) Group average of task sensitivity/ difficulty quantified as slope of the psychometric functions. Units of the horizontal axis are in percent of bar length. Error bars indicate standard errors.

In the *landmark task*, we examined the influence of pixel noise on biases using stimuli of different lengths where only long lines yielded pseudoneglect with short lines producing no leftward biases, if not small numerical trends in the opposite direction. A Pixel Noise (0%/42%/84%) × Task (long vs. short lines) repeated-measures ANOVA produced an effect of Task (F (1, 21) = 17.00, p < 0.001, partial η^2^ = 0.447). Pixel noise had no main effect on biases (F (1.03, 21.56) = 3.54, p = 0.073). Crucially however, the interaction between Noise and Task was significant (F (1.03, 21.56) = 7.76, p = 0.010, partial η^2^ = 0.270), indicating that pixel noise amplifies the difference between long and short lines (Fig 3A). The interaction was mainly driven by an influence of noise in the long line condition as we observed when we submitted the long line data to a follow-up 1-factorial ANOVA with factor Pixel Noise (0%/42%/84%; F (1.03, 21.61) = 8.37, p = 0.008, partial η^2^ = 0.285; significant at a Bonferroni-corrected level of 2.5%). Submitting the short line condition to an equivalent follow-up ANOVA yielded no significant effect (F (1.01, 21.29) = 0.88, p = 0.361). Furthermore, we conducted more detailed post-hoc analyses of the noise effect on long lines by organizing the data into two linear contrasts. However, this merely produced a non-significant trend for the bias at 84% compared to the other noise levels (84% vs. average of 0% and 42%: t (21) = 2.915, p = 0.008, effect size = 0.288; significant after Bonferroni correction). Biases for 0% vs. 42% noise were not significantly different from each other (t (21) = -1.217, p = 0.237). Finally, we contrasted each of the six conditions with zero with one-sample t-tests. This showed that leftward long line biases at all three noise levels were significantly different from zero (0% noise: average PSE = -1.459, SD = 2.011; t (21) = -3.403, p = 0.003, effect size = 0.726; 42% noise: average PSE = -1.096, SD = 1.829; t (21) = -2.810, p = 0.010, effect size = 0.599; 84% noise: average PSE = -6.772, SD = 9.155; t (21) = -3.470, p = 0.002, effect size = 0.739; significant after serial Bonferroni correction). Short line biases were not significantly different from zero (0% noise: average PSE = 0.028, SD = 1.084, t (21) = 0.122, p = 0.904; 42% noise: average PSE = -0.013, SD = 1.155, t (21) = 0.052, p = 0.959; 84% noise: average PSE = 1.300, SD = 6.858, t (21) = 0.889, p = 0.384).

The specific effect of noise on landmark biases could not be explained by task difficulty, because an equivalent ANOVA on the slopes of the psychometric functions produced no comparable Noise ×Task interaction (F (1.56, 32.79) = 0.55, p = 0.538). The significant main effect of Pixel noise (F (1.72, 36.04) = 28.20, p < 0.001, partial η^2^ = 0.590) merely indicated that higher pixel noise levels made the landmark task more difficult (Fig 3B). Also, short lines were slightly more difficult but the effect was not significant (F (1, 21) = 2.89, p = 0.104).

**Fig 4:**
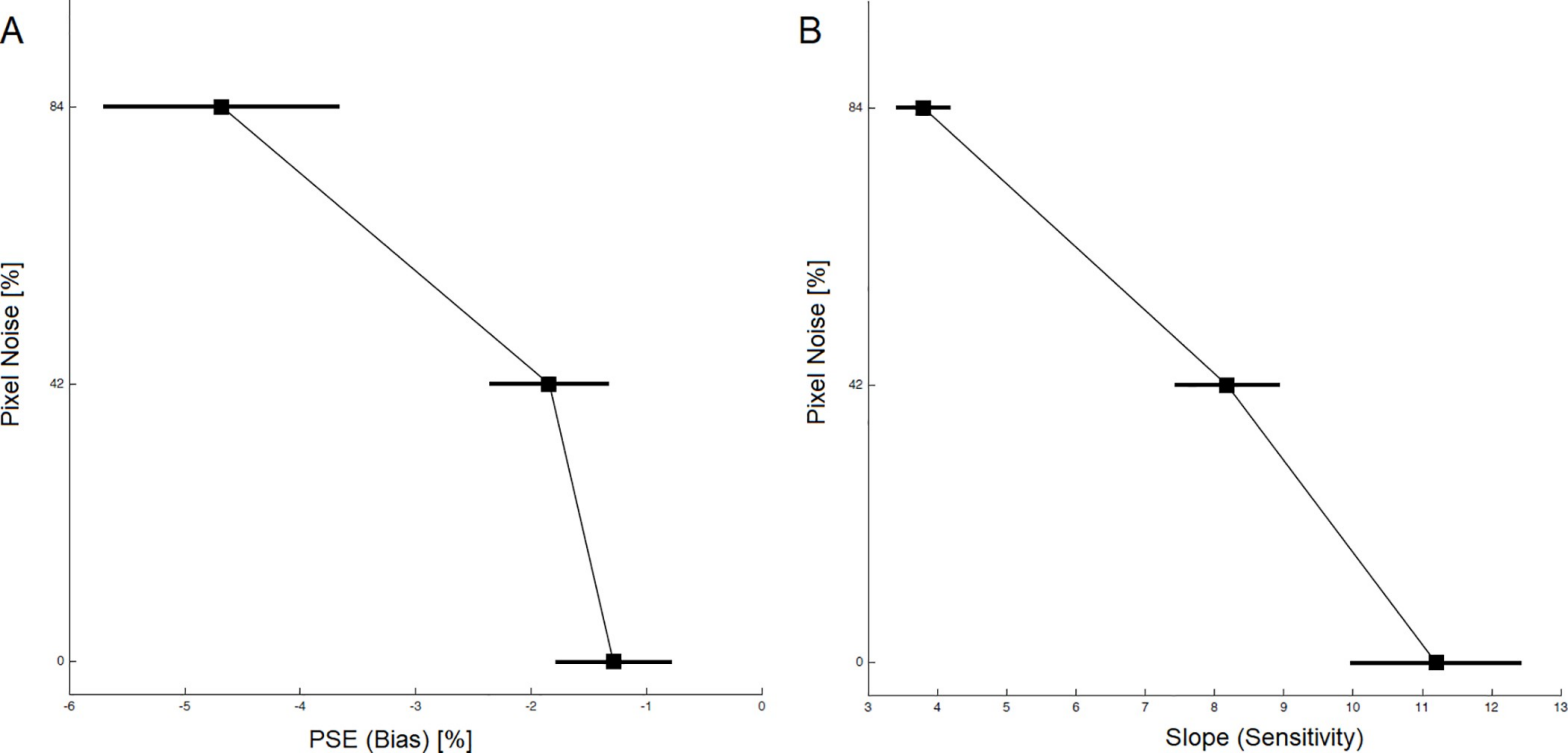
Results for the greyscales experiment. (A) Group average of perceptual bias quantified as point of subjective equality (PSE) of the psychometric functions. (B) Group average of task sensitivity/ difficulty quantified as slope of the psychometric functions. Units of the horizontal axis are in percent of bar length. Error bars indicate standard errors.

Pixel noise also amplified pseudoneglect as measured in the *greyscales task* (Fig 4). A one-way repeated measures ANOVA with factor Pixel Noise (0%/42%/84%) revealed a significant effect (F (1.24, 24.72) = 13.12, p = 0.001, η^2^ = 0.396). Linear contrasts showed that bias at 84% noise was different from the other noise levels (84% vs. average of 0% and 42%: t (20) = 3.790, p = 0.001, effect size = 0.418; significant at a Bonferroni-corrected level of 2.5%), but there was no bias difference between 0% vs. 42% noise (t (20) = 1.676, p = 0.109). Again, one sample t-tests demonstrated that leftward biases at all three noise levels were significantly different from zero after Bonferroni correction (0% noise: average PSE = -1.288, SD = 2.235; t (20) = -2.640, p = 0.016, effect size = 0.508; 42% noise: average PSE = -1.842, SD = 2.289; t (20) = -3.687, p = 0.001, effect size = 0.636; 84% noise: average PSE = -4.643, SD = 0.046; t (20) = -4.643, p < 0.001, effect size = 0.720; significant after serial Bonferroni correction). The one-way ANOVA on slope produced an effect of Noise as well (F (1.63, 32.55) = 33.38, p < 0.001, η^2^ = 0.625), reflecting that the task becomes more difficult with increased pixel noise.

**Fig 5:**
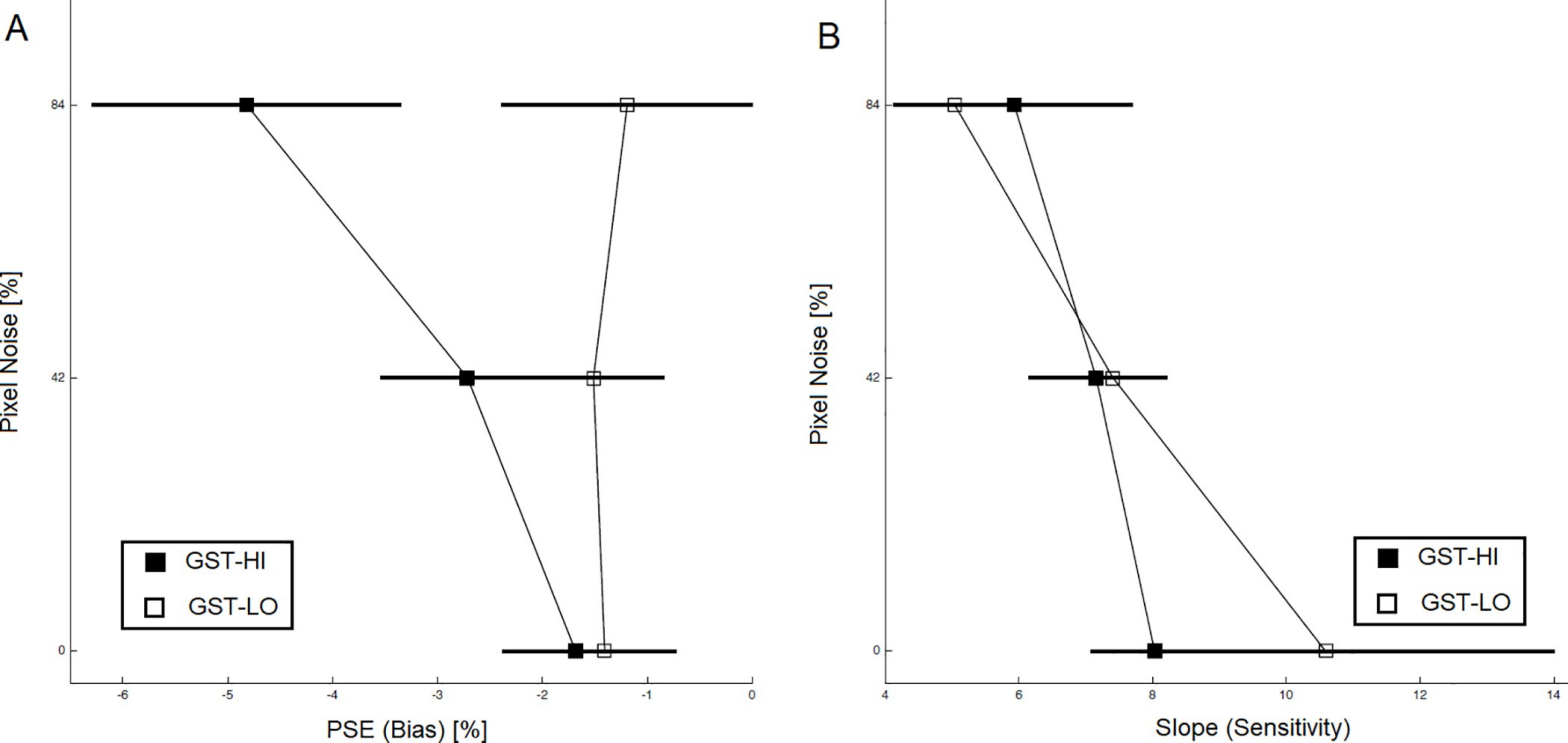
Results for the grating-scales experiment. (A) Group average of perceptual bias quantified as point of subjective equality (PSE) of the psychometric functions. (B) Group average of task sensitivity/ difficulty quantified as slope of the psychometric functions. Units of the horizontal axis are in percent of bar length. Error bars indicate standard errors.

Pixel noise also affected responses in the *grating-scales task* in terms of biases (Fig 5A). Biases submitted to a Pixel Noise (0%/42%/84%) × Task (GST-HI vs. -LO) repeated-measures ANOVA yielded trends for main effects above 5% (Noise: F (1.18, 24.68) = 3.21, p = 0.080; Task: F (1, 21) = 2.45, p = 0.132). Crucially however, the interaction between Noise and Task was significant (F (1.35, 28.31) = 5.72, p = 0.016, partial η^2^ = 0.214), comparable to the interaction in the landmark task. To explore the interaction further, we followed up with one-way ANOVAs at each level of Task. We observed a significant effect of Noise for GST-HI (F (1.24, 26.00) = 8.13, p = 0.006, partial η^2^ = 0.279; significant at a Bonferroni-corrected level of 2.5%) but not for GST-LO (F (1.26, 26.42) = 0.09, p = 0.818). Linear contrasts to compare GST-HI biases revealed significant differences (84% vs. average of 0% and 42%: t (21) = 2.868, p = 0.009, effect size = 0.281; 0% vs. 42%: t (21) = 2.714, p = 0.013, effect size = 0.260; significant after serial Bonferroni correction). Further, one-sample t-tests showed that GST-HI at all three noise levels were significantly different from zero (0% noise: average PSE = -1.683, SD = 3.185; t (21) = -2.479, p = 0.022, effect size = 0.528; 42% noise: average PSE = -2.713, SD = 3.826; t (21) = -3.326, p = 0.003, effect size = 0.709; 84% noise: average PSE = -4.820, SD = 6.826; t (21) = -3.311, p = 0.003, effect size = 0.706; significant after serial Bonferroni correction). GST-LO biases were not significantly different from zero after serial Bonferroni correction (0% noise: average PSE = -1.406, SD = 3.142, t (21) = -2.209, p = 0.048; 42% noise: average PSE = -1.513, SD = 3.071, t (21) = -2.312, p = 0.031; 84% noise: average PSE = -1.191, SD = 5.551, t (21) = -1.007, p = 0.326. Slopes (Fig 5B) submitted to a Pixel Noise × Task repeated-measures ANOVA produced no effects (Noise: F (1.48, 31.09) = 2.66, p = 0.100; Task: F (1, 21) = 0.20, p = 0.659; interaction: F (1.41, 29.77) = 0.66, p = 0.474).

Finally, to compare the effect of pixel noise on pseudoneglect across the three perceptual judgment experiments we calculated confidence intervals for the effect sizes of noise for each of the experimental conditions (long lines: partial η^2^ = 0.285, CI: [0.062, 0.454]; grey scales: partial η^2^ = 0.396, CI: [0.145, 0.552]; GST-HI: partial η^2^ = 0.279, CI: [0.084, 0.420]). This shows that there was no significant difference in effect size, although for the greyscales task a trend for a stronger effect was observed that warrants further investigation in the future.

**Fig 6:**
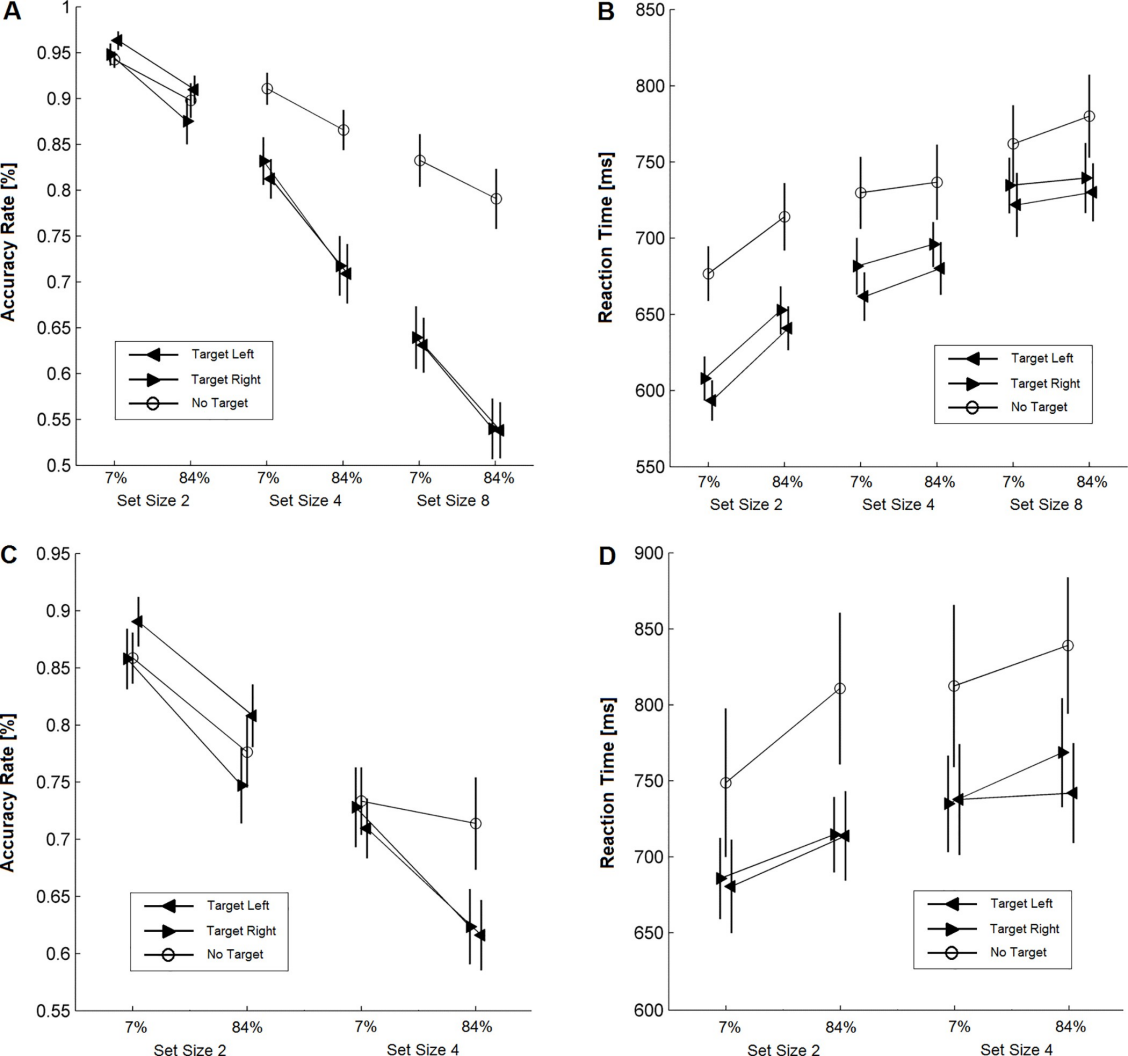
Results for the visual search experiments. (A) Group average of accuracy rates as a function of pixel noise, set size and location of the target presented in the standard visual search task. (B) Group average of median reaction times as a function of pixel noise, set size and location of the target presented in the standard visual search task. (C) Group average of accuracy rates in the masked visual search task. (D) Group average of median reaction times in the masked visual search task. Error bars indicate standard errors.

Unlike perceptual judgments, visual search produced no evidence for an influence of pixel noise on pseudoneglect (i.e., in the form of a 2- or 3-way interaction involving factors Pixel noise and Visual field). Our *standard visual search task* (Fig 6A and 6B) was modelled after a previous paradigm that is known to capture pseudoneglect reliably [16]. We conducted 2 repeated-measures ANOVAs with factors Visual field (target on the left vs. right), Pixel noise (7%, 84%), and Set size (2, 4, 8) to inspect dependent variables accuracy and reaction times, respectively. Accuracy declined with increased Pixel noise (F (1, 24) = 55.73, p < 0.001, η^2^ = 0.699) and Set size (F (2, 48) = 157.24, p < 0.001, η^2^ = 0.868) with only a non-significant trend for a Pixel noise × Set size interaction (F (2, 48) = 2.77, p = 0.080). Visual field had no significant influence (main effect of Visual field: F (1, 24) = 0.022, p = 0.882; Visual field × Set size interaction: F (2, 48) = 1.956, p = 0.155; Visual field × Pixel noise interaction: F (1, 24) = 0.450, p = 0.509; 3-way interaction: F (2, 48) = 0.052, p = 0.924).

Visual field did affect reaction times with faster responses to targets on the left than the right side, consistent with pseudoneglect (F (1, 24) = 5.80, p = 0.024, η^2^ = 0.195). Also, responses were slower with more pixel noise (F (1, 24) = 13.98, p = 0.001, η^2^ = 0.368) and larger set sizes (F (2, 48) = 78.51, p < 0.001, η^2^ = 0.766), and there was a significant Pixel noise x Set size interaction (F (2, 48) = 6.68, p = 0.003, η^2^ = 0.218). However, no other interaction was significant: there was no Visual field × Set size interaction (F (2, 48) = 0.228, p = 0.754). More importantly, Visual field and Pixel noise did not interact (Pixel noise x Visual field: F (1, 24) = 0.153, p = 0.699; Pixel noise x Visual field x Set size: F (2, 48) = 0.003, p = 0.983). In sum, the results of the standard visual search task confirmed previous findings of pseudoneglect during visual search [16] as well as demonstrate that noise increased task difficulty significantly. However, there was no evidence that pixel noise amplified pseudoneglect during visual search in contrast to perceptual judgments.

We continued to try to find an influence of noise on exploratory pseudoneglect; we used a *masked visual search task* where the masks served to disrupt iconic memory, thereby minimizing any possible serial scanning (also see Methods). But again, there was no evidence for pixel noise amplifying pseudoneglect (Fig 6C and 6D): Accuracy yielded main effects of Pixel noise (F (1, 24) = 71.37, p < 0.001, η^2^ = 0.748) and Set size (F (1, 24) = 88.88, p < 0.001, η^2^ = 0.787). Also, there was a Visual field x Set size interaction (F (1, 24) = 12.06, p = 0.002, η^2^ = 0.335), indicating a left visual field advantage for the smaller set size (no main effect of Visual field: F (1, 24) = 1.149, p = 0.294). However, no other interaction was significant: Pixel noise x Visual field: F (1, 24) = 1.056, p = 0.314; Pixel noise x Set size: F (1, 24) = 0.015, p = 0.905; Pixel noise x Visual field x Set size: F (1, 24) = 0.251, p = 0.621).

Likewise, reaction times became longer with increased Pixel noise (F (1, 24) = 6.35, p = 0.019, η^2^ = 0.209) and larger Set size (F (1, 24) = 11.32, p = 0.003, η^2^ = 0.320). But no other main effect or interaction was significant, including any test that involved Visual field: Visual field (F (1, 24) = 0.514, p = 0.481); Visual field x Pixel noise (F (1, 24) = 1.024, p = 0.322); Visual field x Set size (F (1, 24) = 0.542, p = 0.469); Set size x Pixel noise (F (1, 24) = 0.762, p = 0.391); Pixel noise x Visual field x Set size (F (1, 24) = 1.625, p = 0.215). Further, Fig 6D appears to show a trend for a Visual field × Pixel noise interaction when set size was 4. However, a power analysis [82] suggested that for this interaction to become significant, an unrealistically large sample of 680 participants would be required. Further, a paired sample t-test comparing the left vs. right visual field at the noise level of 84% when set size was 4 showed that there was no visual field difference in reaction time (t (24) = -1.311, p = 0.202).

As a last step to ensure that we had not overlooked a pixel noise dependency of pseudoneglect, we revisited the left visual field advantage in terms of accuracy for set size 2 (Fig 6C). We noticed that a similar left visual field advantage had occurred during the standard search task (Fig 6A). Therefore, we submitted the data to an omnibus ANOVA with between-subjects factor Task (standard vs. masked search), and within-subject factors Visual field (target on the left vs. right), Pixel noise (7%, 84%) that selectively focussed on set size 2. Although such selectiveness should bias our odds in favour of finding an influence of pixel noise on pseudoneglect, we did not: There was a significant effect of Visual field (F (1, 48) = 13.313, p = 0.001, η^2^ = 0.217) and of Pixel noise (F (1, 48) = 57.955, p < 0.001, η^2^ = 0.547). The effect of Task was also significant (F (1, 48) = 12.813, p = 0.001, η^2^ = 0.211). Crucially, there was no interaction between Pixel noise and Visual field (F (1, 48) = 2.100, p = 0.154) across 50 participants, and a power analysis suggested that it would require 776 participants to reach significance. No other interaction was significant: Visual field x Task (F (1, 48) = 1.246, p = 0.270); Pixel noise x Task (F (1, 48) = 2.522, p = 0.119); Visual field x Pixel noise x Task (F (1, 48) = 0.081, p = 0.778). This shows that we likely did not overlook any pixel noise dependent effect on search-based pseudoneglect. At the very least, such a noise effect would be substantially smaller than in perceptual judgment tasks.

## Discussion

The aim of the current study was to establish convergent and discriminant validity for pseudoneglect. To this end we tested whether different pseudoneglect measures would be similarly influenced by the same experimental manipulation. Specifically, we manipulated pseudoneglect with pixel noise. We have previously shown that visual images corrupted by pixel noise amplify pseudoneglect biases in the grating-scales task [59] apparently due to visual activation [60] of attentional processes [40]. Therefore, in the current study we tested whether pixel noise has comparable effects on other measures of pseudoneglect. We found that pseudoneglect as measured with the landmark task [67, 71], the greyscales task [12, 13], and the grating-scales task [14], but not pseudoneglect as observed during visual search [16], yielded pseudoneglect biases that were influenced similarly by noise.

Similarities became apparent in that pixel noise increased leftward biases in the experimental conditions of the perceptual judgment tasks: the long lines in the landmark task, the greyscales task, and the high-spatial frequency condition of the grating-scales task. What is more, the noise effect was specific; it did not occur in control conditions that are known to yield no pseudoneglect. Neither the short lines condition in the landmark task nor the low-spatial frequency condition of the grating-scales task showed an influence of pixel noise. Thus, pseudoneglect is not simply the result of an unspecific additive effect. Instead pixel noise appears to target specific aspects of perceptual judgments, at least in the landmark and the grating-scales task. In sum, the observed similarities serve to establish convergent validity. They indicate that perceptual judgment tasks involve specific mechanisms that are similarly affected by pixel noise and that underlie a common form of perceptual pseudoneglect.

A common form of perceptual pseudoneglect is at odds with the frequently held view that perceptual judgment tasks capture many different kinds of pseudoneglect biases as could be concluded because pseudoneglect tasks often are poorly correlated. However, our data offer a different interpretation of the poor correlations; commonalities between perceptual pseudoneglect tasks do seem to exist but are difficult to observe because idiosyncratic, task-specific processes obscure them. It has long been assumed that a core set of mechanisms underlying pseudoneglect exists (e.g., [13, 45]). Our study provides important direct support for this assumption to be true in regards to perceptual forms of pseudoneglect, despite their poor correlations with one another.

In contrast, we found no evidence that exploratory forms of pseudoneglect as observed during visual search would share the same core mechanisms. That is, we found no evidence for a commonality between exploratory pseudoneglect and perceptual pseudoneglect in that pixel noise did not increase exploratory pseudoneglect biases, thereby further establishing discriminant validity (aside from the control conditions in the perceptual judgment tasks that also demonstrate the specificity of the noise effect). We have three reasons to assume that we did not overlook such a noise effect during visual search. First, the search paradigm that we used is well understood as to which of its parameters will reliably give rise to pseudoneglect [16], and indeed, we did observe pseudoneglect. Pseudoneglect surfaced in different dependent variables in our main, standard search experiment vs. the follow-up experiment where we used masks. However, that is not surprising given the substantial impact visual masks have on task difficulty and iconic memory.

Second, our experimental manipulation of the searches with pixel noise was sufficiently strong, and the experiments had sufficient power; noise had a main effect on search performance which suggests that difficulty increased in a manner comparable to the perceptual judgment tasks (see effect sizes of noise on the slopes of the psychometric functions). Next, we only used two levels of noise which should have made it easier to detect a noise effect on pseudoneglect compared to the perceptual judgment experiments where we used 3 noise levels. In addition, there was no risk of a floor effect because in most conditions participants performed well above chance and further because the moderate effect size of exploratory pseudoneglect (i.e., the main effect of visual field) would not have hindered the amplifying influence of noise on pseudoneglect biases (i.e., a noise by visual field interaction). Finally, we argue that the number of 50 participants across the two search experiments was sufficient compared to other experiments (21 or 22 participants in the current perceptual judgment tasks, 17 participants in [16]). Our analyses suggest that it would have been necessary to test more than 10 and up to 50 times as many participants to find a significant effect of noise. Even so, such a small noise effect would be qualitatively different from the noise effect observed during perceptual judgments.

Third, we can be quite certain that the two search paradigms that we tested here maximized our chances of detecting an influence of pixel noise on pseudoneglect because they likened the perceptual judgment tasks in several important ways with relatively similar stimuli, similar total test time, and comparable limitation in presentation times. Further research is necessary to test whether self-terminating searches with or without eye movements yield noise-sensitive pseudoneglect. However, once again such a noise effect would be different from the noise effect observed during perceptual judgments.

Thus, our results suggest that there are at least two different groups of pseudoneglect tasks that do–or do not–share a sensitivity to pixel noise. Thus, the two groups of pseudoneglect tests do not, at least to some extent, seem to share the same underlying core mechanisms. Aside from a perceptual form of pseudoneglect, another kind is different and could involve exploratory functions, although more research will be required to establish exploratory pseudoneglect as a cohesive entity. Nevertheless, for now it is interesting to note that our conclusion agrees with neuropsychological findings in patients with spatial neglect where relatively independent perceptual and exploratory/ visuomotor deficits have been proposed [55–57]. Crucially, the findings in the current study suggest that dissociable deficits in patients map onto corresponding groups of functions in healthy people, despite the methodological pitfalls that come with neuropsychological data. Another attempt to conceptualize functions and dysfunctions in healthy individuals and patients arrives at similar conclusions, arguing that functions associated with neglect and pseudoneglect might cluster into sensorimotor and perceptual processes, respectively, depending on how strongly biases are modulated by prism adaptation [48]. More research will be required to understand the relationship between prism adaptation and pixel noise, as well as between sensorimotor/ exploratory vs. perceptual forms of pseudoneglect.

The current findings, however, offer some insights into the mechanisms that may or may not underlie perceptual pseudoneglect. It seems we can rule out global attention because visual searches, just like perceptual judgments, require people to distribute attention widely across both visual hemi-fields and simultaneously–at least during our masked searches.

Also, our data are inconsistent with mechanisms outlined in the activation-orientation hypothesis [71]. The hypothesis proposes that each hemisphere generates a contralateral attentional bias and that these biases, governed by sensory stimulation, create an attentional imbalance between the left and right hemisphere due to the predominance of one hemisphere, thereby determining a net attentional bias that is reflected in a directional vector of attentional orienting [83]. However, we found no evidence for stronger leftward orienting during visual search fuelled by noise-based activation (for additional limitations of the hypothesis see [59, 84]).

Next, several paradigmatic differences between perceptual judgments and visual search as well as their associated functions also appear to be unrelated to pixel noise-sensitive pseudoneglect. First, it is unlikely that pixel noise interacts with preattentive or “bottom-up” aspects of perception during perceptual judgments. That is, these tasks involve stimuli whose parts could be spotted easily, i.e., the left and right halves of the landmark lines, the black regions of the greyscales and the high-spatial frequency components of the grating-scales. In contrast, visual search targets as used here do not “pop out.” Crucially however, pop-out during perceptual judgments must have been much reduced when stimuli were very noisy [85]. It seems unlikely that minimal “pop-out” would yield maximum pseudoneglect biases. As a second difference in paradigms, perceptual judgments are discrimination tasks whereas our visual search tasks required target detection. However, precisely such target detection, not discrimination, yields left-visual field advantages consistent with pseudoneglect [16]. Third, visual search arrays cover a much larger vertical portion of the visual field compared to stimuli in perceptual judgment tasks. Extension of perceptual judgment stimuli along the vertical axis might diminish pseudoneglect biases. For example, landmark biases decline with more rectangular shaped stimuli compared to line-shaped stimuli [5]. But it is unclear why search biases, even if reduced, should be insensitive to noise.

Instead, we speculate that perceptual pseudoneglect might be associated with right-dominant mechanisms used to integrate visual information across hemispheres. Little interhemispheric information integration is necessary for visual search tasks. The brain can decide whether a target is present or absent in each visual field separately, and then with minimal bandwidth integrate the information across hemispheres. Because the corpus callosum constitutes a significant bottleneck, it is likely that the brain does indeed use such a data pre-processing strategy for visual search. In contrast, for perceptual judgments visual information from the left and right visual fields first needs to be compared and integrated before any binary decisions can be made. It follows that perceptual judgments need more interhemispheric bandwidth, and they need more capacity to compute a decision. To this end, perceptual judgments might rely on a memory buffer for online visuo-spatial computations that is mainly lateralized in one hemisphere. Further, imperfections in this buffer would result in perceptual pseudoneglect.

In conclusion, using pixel noise as a tool to identify commonalities between different manifestations of pseudoneglect, we propose a common form of perceptual pseudoneglect that is different from other kinds of pseudoneglect, apparently to do with exploratory functions. Additional studies are required to scrutinize other pseudoneglect tasks so as to expand and identify the different constructs of pseudoneglect. Here, a classic approach of correlation studies might be helpful provided that (a) sufficiently large groups of participants are recruited to compensate for small inter-task covariance, and (b) the employed test batteries comfortably outnumber the expected number of extracted factors. Still, these factors will likely require additional studies using various forms of experimental manipulations of the different pseudoneglect biases. A visuo-spatial iconic buffer as the correlate of the perceptual form of such biases should be the subject of future investigations.

## Supporting information

S1 File ChenNiemeier_NoisePseudoneglect_Data; spreadsheet containing individual participant data. Tab “Landmark” contains the data for the landmark test. Columns B-D: Biases for long lines and 0%/42%/84% noise. Columns E-G: Biases for short lines and 0%/42%/84% noise. Columns H-J: Slopes for long lines and 0%/42%/84% noise. Columns K-M: Slopes for short lines and 0%/42%/84% noise. Tab “Greyscales” contains the data for the greyscales task. Columns B-D: Biases for stimuli with 0%/42%/84% noise. Columns E-G: Slopes for stimuli with 0%/42%/84% noise. Tab “GST” contains the data for the grating-scales task. Columns B-D: Biases for the HI condition and 0%/42%/84% noise. Columns E-G: Biases for the LO condition and 0%/42%/84% noise. Columns H-J: Slopes for the HI condition and 0%/42%/84% noise. Columns K-M: Slopes for the LO condition and 0%/42%/84% noise. Tab “VisualSearch1” contains the data for the first visual search task. Columns B-S: accuracy data. Columns T-AK: reaction time data. Tab “VisualSearch2” contains the data for the second visual search task. Columns B-M: accuracy data. Columns N-Y: reaction time data.
(XLSX)

S1 Appendix Data4PLOSone. Raw data of all experiments in Matlab data format. The files can be downloaded here: https://www.dropbox.com/sh/squbgcdpz9ysgyj/AAC-K5GS2zLN5kWNr4kcB124a?dl=0.
(ZIP)

S2 Appendix Programs4PLOSone. Matlab programs for data analysis. The files can be downloaded here: https://www.dropbox.com/sh/btpcjl14t7eq6ob/AABwmermox5gp5hfa0BoD-zOa?dl=0.
(ZIP)
